# Neurological and Psychiatric Sequelae of Developmental Exposure to Antiepileptic Drugs

**DOI:** 10.3389/fneur.2012.00182

**Published:** 2012-12-27

**Authors:** Evan R. Gedzelman, Kimford J. Meador

**Affiliations:** ^1^Department of Neurology, Emory University School of MedicineAtlanta, GA, USA

**Keywords:** antiepileptic, neurodevelopmental, neuronal plasticity, teratogenicity, fetal, neurotransmitter

## Abstract

The neurons in the developing mammalian brain are susceptible to antiepileptic drug (AED) effects. It is known that later in life deficits in cognitive performance as well as psychiatric deficits can manifest after early AED exposure. The extent of these deficits will be addressed. This review will attempt to draw parallels between the existent animal models and human studies. Through analysis of these studies, important future research will be elucidated and possible new and emerging therapies will be discussed.

## Introduction

The developing brain is exquisitely sensitive to drug-induced perturbations of the chemical environment of neurons. Exposure to these agents during mammalian brain maturation has been associated with alterations in cognitive performance later in life including risk factors for neuropsychiatric outcomes. The extent to which the preclinical animal findings translate to the human data will be discussed.

The time course of brain development in experimental animals and the manner in which that time course maps onto brain development in humans are important to research design and interpretation of findings. Neurological and psychiatric abnormalities can occur following developmental exposure to antiepileptic drugs (AEDs). Unfortunately, the sensitive periods for toxicity to various drugs are not certain in humans. Research on fetal alcohol syndrome may be able to provide a vital link to inform studies on AEDs. The mechanisms by which early life drug exposure alters nervous system function in later developmental phases will be discussed.

Through animal models and through analysis of clinical outcomes in humans the current state of knowledge and key targets for future research can be elucidated. It is important to evaluate novel therapeutic paradigms to minimize AEDs effect on the developing brain, while maximizing clinical benefit.

## Antiepileptic Drug Studies in Humans

### Carbamazepine

No effect of fetal carbamazepine exposure on IQ was reported in a prospective, observational, evaluator-blinded study with matched controls (Scolnik et al., 1994). In a study of preschool children, no adverse effects of fetal carbamazepine exposure were seen in this population-based, longitudinal follow-up study (Wide et al., 2002). No IQ difference was found in a retrospective, blinded study controlling for maternal IQ in 52 children of women with epilepsy (WWE) exposed to carbamazepine versus 80 unexposed children (Adab et al., 2004). No difference in IQ was seen in 86 children of WWE exposed to carbamazepine monotherapy versus 45 children of WWE on no AED or 141 healthy control children in a prospective, population-based, blinded, observational study controlling for maternal education (Gaily et al., 2004). When data from this study were pooled with data from another prospective population-based study, mental retardation occurred in only 1 of 84 children exposed to polytherapy (Gaily et al., 1988, 2004). Again, no IQ difference was seen in children of WWE exposed to carbamazepine versus no AED in a population-based, evaluator-blinded study (Eriksson et al., 2005). Evaluation on autism spectrum disorders in 80 children exposed to carbamazepine monotherapy showed no difference in frequency from the general population in a retrospective population-based study (Rasalam et al., 2005). In a prospective observational multicenter study at ages 3 and 4.5 years, children exposed *in utero* to carbamazepine did not differ from lamotrigine or phenytoin, however, they had better IQ outcomes than valproate (Meador et al., 2009, 2012). Follow-up investigations showed fetal exposure to carbamazepine as well as lamotrigine, phenytoin, and valproate were associated with a reduction in verbal compared to non-verbal abilities at age 3 and 4.5 years (Meador et al., 2011, 2012). This finding requires replication in separate cohorts to determine if language abilities are particularly susceptible to fetal AED exposure.

### Lamotrigine

Children exposed *in utero* to lamotrigine at ages 3 and 4.5 years did not differ from those exposed to carbamazepine or phenytoin, but had better IQ outcomes than those exposed to valproate at age 3 and 4.5 years in a prospective observational multicenter study (Meador et al., 2009, 2012). In a follow-up investigation in the same cohort, dose-related effects on verbal and non-verbal cognitive measures were assessed in fetal AED exposed 3-year-old children (Meador et al., 2011). This investigation set out to ascertain whether differential long-term neurodevelopmental effects exist across four commonly used AEDs (valproate, carbamazepine, lamotrigine, and phenytoin). The Differential Ability Scales, Preschool Language Scale, Peabody Picture Vocabulary Test, and Developmental Test of Visual-Motor Integration were utilized to calculate cognitive outcomes in 216 children. Children exposed *in utero* to each drug exhibited lower verbal abilities than non-verbal. A dose effect was not seen for lamotrigine or phenytoin at age 3 (Meador et al., 2011).

### Levetiracetam

In a recent study comparing levetiracetam, valproate, and a control group at less than age 24 months, children exposed to levetiracetam obtained higher developmental scores compared with children exposed to valproate (*n* = 51 levetiracetam; *n* = 44 valproate; *p* < 0.001). The levetiracetam group did not differ from the control group (*n* = 97; *p* = 0.62; Shallcross et al., 2011). Limitations of the study included young age at assessment, retrospective data for seizures and alcohol/tobacco use, and completer rates of only 37% for valproate and 58% for levetiracetam. Given these limitations and the fact that this is the only study on neurodevelopment effects of levetiracetam, caution should be exercised when utilizing this source for clinical application. Replication of the findings is needed.

### Phenobarbital

No difference in IQ was seen in 35 children of WWE exposed *in utero* to phenobarbital monotherapy versus 4,705 children of mothers without epilepsy according to a study done in Shapiro et al. (1976). In contrast, two studies found approximately 0.5 SD lower verbal IQ scores in the men exposed to phenobarbital. These studies involved separate cohorts of 114 adult men of mothers without epilepsy, exposed *in utero* to phenobarbital (Reinisch et al., 1995). It has also been shown that young children exposed to phenobarbital have statistically different IQ scores than control groups. One investigation of phenobarbital exposure in young children with febrile seizures offers insights into the effects of phenobarbital on the immature brain. A total of 217 children with febrile seizures were randomized to a phenobarbital group or a placebo group, and tested 2 years later; those exposed to phenobarbital had lower IQ (Farwell et al., 1990). If possible, avoidance of phenobarbital in WWE during pregnancy, may be considered to reduce the risk of poor cognitive outcomes (Harden et al., 2009).

### Phenytoin

No effect of fetal phenytoin exposure on IQ was seen in two prospective, blinded, population-based studies that controlled for socioeconomic class or maternal educational level (Shapiro et al., 1976; Gaily et al., 1988). Another study using the same design but drawn from a different patient pool demonstrated lower IQ at age 7 years in children of WWE exposed to phenytoin versus healthy control children of mothers without epilepsy (Hanson et al., 1976). Slightly lower scores for locomotor development were reported in phenytoin-exposed children versus fetal carbamazepine-exposed children and unexposed children in a population-based longitudinal follow-up study of preschool children (Wide et al., 2002). Phenytoin-exposed children had significantly lower IQ scores in a blinded-evaluator, case–control study (Vanoverloop et al., 1992). One prospective, blinded observational study demonstrated lower IQ for children with fetal phenytoin exposure compared with matched control (Scolnik et al., 1994), however no effect was seen when maternal IQ was taken into consideration (Loring et al., 1994). In a recent prospective observational multicenter studies of monotherapy, children at age 3 and again at age 4.5 years exposed to phenytoin *in utero* had better IQ outcomes than those exposed to valproate but did not differ from those exposed to carbamazepine or lamotrigine (Meador et al., 2009, 2012). The AAN recommended avoiding phenytoin in WWE during pregnancy based on level C evidence (Harden et al., 2009), however more conclusive determinations on the risks of phenytoin will require additional studies.

### Valproate

Two prospective, population-based, evaluator-blinded studies in children of WWE exposed *in utero* to valproate monotherapy compared with children with carbamazepine monotherapy or no AED exposure demonstrated lower verbal IQ or full-scale IQ in the valproate group (Gaily et al., 2004; Eriksson et al., 2005). Unfortunately, these studies are limited by the sample size of the valproate monotherapy group (*n* = 26 across the two studies). Additionally, the studies revealed significantly lower maternal education or IQ in the valproate group compared with other WWE, which could have confounded the results. One retrospective study showed an increased need for special education in children exposed to valproate monotherapy compared with other monotherapies or the unexposed group (Adab et al., 2001). Another large retrospective investigation from the same center, which controlled for maternal IQ, demonstrated lower verbal IQ (about 10 points) in children exposed *in utero* to valproate (*n* = 41) versus other monotherapy groups and an unexposed group (Adab et al., 2004). The decrease in IQ in the valproate group was dose-dependent.

A prospective, observational, evaluator-blinded, multicenter study, which enrolled pregnant WWE on AED monotherapy controlling for a variety of potential confounding factors including maternal IQ, showed children exposed *in utero* to valproate had lower IQ at ages 3 (7–9 points) and 4.5 (8–11 points) years compared with other AEDs (carbamazepine, lamotrigine, or phenytoin; Meador et al., 2009, 2012). The detrimental effect of valproate on IQ was dose-dependent. According to another analysis of the same cohort, children exposed *in utero* to valproate monotherapy had reduced verbal and non-verbal cognitive outcomes versus carbamazepine, lamotrigine, and phenytoin. The impact of valproate appeared greater on verbal than non-verbal abilities, and the effect was dose-dependent (Meador et al., 2011).

In another prospective study, the early cognitive development of children of WWE (*n* = 198) was compared to general population controls (*n* = 230). Children under 2 years old were assessed by a blinded-evaluator using the Griffiths Mental Development Scales. There was a statistically significant increased risk of delayed early development in the group exposed to valproate compared to controls. Children exposed to carbamazepine or lamotrigine *in utero* did not differ from the control group (Bromley et al., 2010).

An observational cohort study was conducted in children from Northern Ireland aged 9–60 months born to mothers enrolled in the UK Epilepsy and Pregnancy Register (Cummings et al., 2011). Neurodevelopment was evaluated using either the Bayley Scales of Infant Development or the Griffiths Mental Development Scales. 210 children were assessed by a single researcher blinded to AED exposure. There was evidence of mild or significant developmental delay in 23 (39.6%) children exposed *in utero* to valproate, 10 (20.4%) exposed to carbamazepine, and 1 (2.9%) exposed to lamotrigine compared to two (4.5%) children in the control group. Multivariable analysis demonstrated that *in utero* exposure to valproate (OR 26.1, 95% CI 4.9 to 139; *p* < 0.001) and carbamazepine (OR 7.7, 95% CI 1.4 to 43.1; *p* < 0.01) had a significant detrimental effect on neurodevelopment, but no adverse effects were seen for lamotrigine.

A recent study from Australia (Nadebaum et al., 2011), evaluated IQ in school-aged children exposed to valproate and polytherapy during pregnancy (using the Wechsler Intelligence Scale for Children – fourth edition). The results suggested that there is a dose-dependent negative impact on verbal intellectual abilities in the group exposed to valproate, and that valproate may also affect working memory. Additionally, the possibility that inclusion of valproate in polytherapy regimens may underlie reduced mean scores was discussed (Nadebaum et al., 2011).

One prospective and two retrospective studies have reported an increased risk for autistic spectrum disorder or behavioral abnormalities in valproate-exposed children (Rasalam et al., 2005; Bromley et al., 2008; Vinten et al., 2009). Avoiding valproate in WWE during pregnancy, if possible, should be considered to reduce the risk of poor cognitive and verbal outcomes (Harden et al., 2009).

### Benzodiazepines

The risks of fetal exposure from benzodiazepines to cognition and behavior are unknown secondary to a dearth of adequate data in human studies.

### Other antiepileptic drugs

The risks of fetal exposure from other AEDs to cognition and behavior are unknown secondary to a dearth of adequate data in human studies.

### Polytherapy

According to one prospective study done in the 1970s (Shapiro et al., 1976), no difference in IQ was noted among children exposed to phenytoin and phenobarbital polytherapy versus other children of WWE. However, subsequent prospective studies have found otherwise. Children exposed to AED polytherapy *in utero* had lower cognitive scores than healthy controls and children of WWE exposed to monotherapy (Losche et al., 1994). Another study showed children exposed *in utero* to AED polytherapy had impaired verbal and non-verbal IQ compared with children exposed to monotherapy (Koch et al., 1999; Gaily et al., 2004). A recent population-based study utilized The Swedish School Mark Registry to evaluate the long-term neurodevelopmental effects of children of WWE exposed to AEDs *in utero* (Forsberg et al., 2011). Data was obtained about school performance from the last year of compulsory school, at age 16. Medical records of 1,235 children were analyzed. 641 children had AED exposure *in utero* in monotherapy, 429 in polytherapy, and 165 no known AED. School grades in four subjects were analyzed: sports, math, English, and Swedish. For each subject grades were in four groups: no grade (which means that the child had not attended the lectures in that subject enough to get a grade), not passed, passed, and passed with excellence. Children born to WWE had a decreased chance of getting a “pass with excellence.” The monotherapy group, mainly those exposed to carbamazepine or phenytoin, did not have a significantly increased risk of not receiving a final grade (OR = 1.10; 95% CI 0.79–1.80). Notably, the polytherapy group had an increased risk of not receiving a final grade [i.e., graduating; OR = 2.99; 95% confidence interval (CI) 2.14–4.17; Forsberg et al., 2011]. If possible, AED polytherapy should be avoided in WWE to reduce the risk of poor cognitive and verbal outcomes. However, the risk of polytherapy combinations comprised of the newer AEDs is unknown.

## Severity of Seizure Disorder

In WWE, the severity of the seizure disorder, including whether or not a patient’s particular seizures are generalized tonic-clonic seizures, could confer increased risk to the fetus for adverse outcomes. Of necessity, this leads to use of higher doses of AEDS and even polytherapy during pregnancy. In turn, this could lead to increased risk for cognitive deficit. However, even though the severity of the seizure disorder can affect fetal outcome, the effects of AEDs on the child’s cognitive outcome cannot be explained by this factor as it was assessed in the analysis for the Neurodevelopmental Effects of Antiepileptic Drugs (NEAD) study. One study reported that >5 convulsions reduced cognitive outcomes (Adab et al., 2004), but additional research is needed.

## Comparison of Animal and Human Studies

Antiepileptic drugs can produce both anatomical (i.e., major congenital malformations, MCMs) or behavioral (i.e., cognitive) teratogenicity in humans. The exact mechanisms are uncertain, but current hypotheses include folate deficiency, ischemia, neuronal suppression, reactive intermediates (e.g., free radicals or epoxides), and AED-induced neuronal apoptosis with associated dysfunction in surviving neurons (Meador et al., 2011). Because the highest risk of MCMs is from first-trimester AED exposure, but the highest risk of behavioral defects appears to be primarily from third-trimester exposure, it seems reasonable to assume that the mechanisms underlying anatomical and behavioral teratogenesis are probably different.

The leading hypothesis for the mechanism behind behavioral/cognitive dysfunction involves AED-induced apoptosis and associated dysfunction in surviving neurons. Genetic predisposition likely plays a role and could involve interaction of teratogens with multiple-liability genes (Finnell et al., 1987). This, in part, may explain the observed individual variability.

In animal studies, alcohol has been shown to produce widespread neuronal apoptosis subsequently leading to neurobehavioral deficits in the developing brain (Ikonomidou et al., 2000). Neuronal dysfunction is also found in the remaining neurons (Medina et al., 2003). The neurotoxic effects of ethanol on the human fetal brain (i.e., fetal alcohol syndrome) have been recognized for almost four decades. Of note, a single episode of ethanol exposure of several hours duration can trigger a massive wave of apoptotic neurodegeneration in the developing rat or mouse brain (Olney et al., 2002). The window of vulnerability coincides with the developmental period of synaptogenesis, which is estimated in humans to occur from the sixth month of gestation to several years after birth (the so-called brain growth-spurt period). *N*-methyl-d-aspartate (NMDA) antagonist and gamma-aminobutyric (GABA) mimetic traits of ethanol may be responsible for its apoptogenic action, as other drugs that block NMDA glutamate receptors or mimic GABA at GABA(A) receptors also trigger apoptosis in the developing brain. This is clinically significant because many agents used therapeutically in humans have NMDA antagonist or GABA mimetic properties including barbiturates, benzodiazepines, and many other anticonvulsants (Olney et al., 2002).

Fetal alcohol syndrome is a major cause of neurodevelopmental deficits. These deficits may result from disruption of neuronal plasticity and neocortex development. During the third-trimester equivalent of human gestation, alcohol exposure may have severe and enduring consequences on sensory processing and learning as this is when the functional properties and connectivity of neocortical neurons start to develop. One study used the monocular deprivation model of neural plasticity in ferrets, as it shares many common mechanisms with learning (Medina et al., 2003). This study demonstrated that a brief period of alcohol exposure during development in the immature brain impairs ocular dominance plasticity later in the ferrets’ life. This model provides a novel approach to investigate and elucidate how alcohol disrupts neural plasticity (Medina et al., 2003). In addition, ferrets were exposed to alcohol on alternate days for 3 weeks leading to discontinuous levels of blood alcohol that still induced reduced cortex plasticity. This suggests that reduced cortex plasticity could be due to a peak dose effect and not just a continuous effect of total alcohol exposure. As mentioned above, a single episode of ethanol exposure of several hours duration can trigger a massive wave of apoptotic neurodegeneration in the developing rat or mouse brain (Olney et al., 2002). So, it follows that neuronal dysfunction may be present in some of the residual neurons even after a single exposure. Evidence for the importance of peak versus total exposure for AEDs has been seen in preliminary animal data (see below), but has yet to be addressed in human studies.

Recent animal testing has focused primarily on apoptosis; now there is need for more cognitive and physiological testing in animals to parallel studies in humans. One of the first studies to assess the effects of AEDs in animals for behavioral changes was done in Vorhees (1985), which evaluated behavioral teratogenicity in rats using multiple AEDs including diphenylhydantoin, trimethadione, and phenobarbital. Another landmark animal study done in Phillips and Lockard (1993) demonstrated that infant primates exposed to phenytoin, in monotherapy or in polytherapy with stiripentol led to increased risk for hyperexcitability (screeching, refusing to attend to stimuli, lack of visual orientation). In another study by the same investigators, they found that infant monkeys exposed to carbamazepine monotherapy or carbamazepine + stiripentol polytherapy did not demonstrate increased risk for hyperexcitability (Phillips and Lockard, 1996). These trailblazing studies are of paramount importance because they provide an early example of an approach to assessment of behavioral teratogenesis of AEDs in animal models. Further, they informed and inspired subsequent investigations in humans. More studies in this vein would allow us to draw parallels between the animal and human exposures to AEDs and thus direct therapy more appropriately in humans.

The findings of alcohol-induced apoptosis in fetal animal brain led to the use of the apoptotic model in studies with AEDs in neonatal rats. Neonatal rats have been used in AED studies because their developmental period parallels the human third-trimester developmental period. Thus, this model is likely to reflect the susceptibility period to AEDs’ adverse effects in humans. AEDs that have been shown to produce widespread neuronal apoptosis in the neonatal rat brain include phenytoin, vigabatrin, valproate, clonazepam, diazepam, and phenobarbital (Bittigau et al., 2002, 2003; Asimiadou et al., 2005; Stefovska et al., 2008; Ikonomidou and Turski, 2010). The effect can occur with single dose exposure, is dose-dependent, and occurs at therapeutically relevant blood levels. Further, two of these AEDs given at below threshold dosages, can still trigger the full apoptotic response, suggesting a synergistic effect. Additionally, it is evident that valproate induces neuronal apoptosis at blood levels which are relatively lower in regards to valproate’s therapeutic range compared to other AEDs (Bittigau et al., 2003). This may explain why cognitive deficits are seen more commonly in humans after fetal exposure to valproate. Some AEDs that do not produce apoptosis in monotherapy can enhance apoptosis induced by another AED (Katz et al., 2007). These include topiramate, carbamazepine, or lamotrigine (Glier et al., 2004; Manthey et al., 2005; Kim et al., 2007). This suggests that polytherapy could increase the risk. Levetiracetam is the only AED of those tested to date that does not produce apoptosis in monotherapy or enhance apoptosis of other AEDs (Manthey et al., 2005). Unfortunately, most AEDs have not been tested in this model. Animal experiments like these naturally raise a red flag that some commonly utilized AEDs could have similar adverse effects in children of WWE exposed *in utero* or as neonates, however, clinical studies are needed to confirm if similar effects occur in the human brain.

Antiepileptic drugs target neurotransmitters, ion channels, and second messenger systems in the brain. These targets regulate processes essential not only for propagation of seizures but for learning, memory, emotional behavior, and brain development. The mechanisms that explain adverse effects of AEDs are not fully understood but include interference with neurogenesis, axonal arborization, synaptogenesis, cell proliferation and migration, synaptic plasticity, and apoptosis (Ikonomidou and Turski, 2010).

Antiepileptic drugs alterations to neurotransmitter systems could potentially induce teratogenesis. This can disrupt both neuronal migration and proliferation. Some AEDs can inhibit glutamate action, e.g., topiramate and felbamate. GABA agonists include phenobarbital, benzodiazepines, and valproate. The neurotransmitter medium in the developing brain plays a large role in the regulation of neuronal differentiation and migration. Based on animal studies, enhanced GABA inhibition and blockade of NMDA receptors impairs neurogenesis and cell migration (Manent et al., 2007; Stefovska et al., 2008). This can lead to cortical dysplasias and reduced brain volume. Enhanced GABA activity (Wong and Wong, 2001) and drug-induced inhibition of NMDA receptor activity (Ogura et al., 2002) can also disrupt synaptogenesis. In children exposed *in utero* to AEDs, these neurotransmitter changes and resultant cellular changes may account for impaired cognition (Palac and Meador, 2011).

Antiepileptic drugs alterations of genetic factors could affect plasticity-related genes as a possible mechanism leading to behavioral teratogenesis. One study (Roullet et al., 2010) addressed this in a model where mouse pups were exposed *in utero* to valproate. Physical development, olfactory discrimination, and social behavior was measured. The exposure resulted not only in motor and sensory deficits, but dysfunctional social behavior as well. Brain derived neurotrophic factor (BDNF) and NMDA receptor subunits NR2A and NR2B were assessed. *In situ* hybridization revealed lower cortical expression of BDNF mRNA in the valproate-exposed animals. The results suggest that alterations in plasticity-related genes may lead to this behavioral phenotype. Further, valproate-treated mice displayed physical development delays, behavioral abnormalities, and modifications at the level of gene expression reported in autism. This finding opens up the possibility to examine specific factors like BDNF in the development of autistic-related traits (Roullet et al., 2010).

It may well be that dysfunction in the surviving neurons contributes in a greater fashion to impaired cognition than neuronal apoptosis itself; for example, young children suffering infantile hemiplegia can develop normal language function despite severe abnormalities in their dominant hemisphere because of normal plasticity in the remaining non-dominant hemisphere. In fact, a recent animal study (Forcelli et al., 2012a) demonstrated that exposure to AEDs during a sensitive postnatal period impairs synaptic maturation in neurons that survive the initial drug exposure. This suggests a mechanism by which early AED exposure can lead to impaired cognitive and behavioral outcomes and alerts us to the necessity of identifying therapies that control seizures but do not compromise synaptic maturation.

## Needed Future Research

Five areas for future research are highlighted.

### 1 Additional AED testing

All AEDs need to be tested in animal models as well as in human investigations. Future research needs to link animal and human studies more effectively. Morphological changes as well as functional deficits in animals should be related to human outcomes to better understand how animal data can guide investigations in humans and the use of AEDs in treatment of women of childbearing age. As mentioned before, recent animal testing has focused primarily on apoptosis; now there is need for more cognitive and physiological testing in animals to parallel studies in humans. More studies of this type are needed similar to the early investigations assessing the effects of AEDs in animals for behavioral changes (Vorhees, 1985). Of note, early investigations such as the one mentioned above provided the chief impetus for the genesis of the ongoing NEAD study in humans.

A recent animal study on the long-term impact of AED exposure during brain development examined phenobarbital, phenytoin, and lamotrigine (Forcelli et al., 2012b). Neonatal AED exposure resulted in adult deficits in spatial learning in the Morris water maze and decreased social exploration for all three AEDs. However, there were a range of different deficits particular to each drug seen in addition to the shared deficit. These results suggest that AED exposure during a small window in the neonatal period is enough to lead to a range of behavioral deficits later in life. Additionally, the variance of subsequent behavioral deficits seen across the three drugs in this study suggests that the specific profile of behavioral deficits varies across AEDs. Of note, the behavioral differences seen across these three AEDs were not predicted by each drug’s mechanism of action nor by the proapoptotic effect of the drugs. The findings suggest that AED exposure during the early developmental period of humans should be viewed as a risk factor for impaired cognitive and psychiatric outcomes later in life. Investigations in humans are limited to only a few AEDs. Additional studies are needed to establish the long-term effects of fetal AED exposure across a range of cognitive and behavioral domains.

### 2 AED polytherapy

The impact of polytherapy including specific combinations on cognitive outcomes is unclear. Polytherapy for both older and newer AEDs needs to be systematically evaluated in animals and humans.

### 3 Pharmacokinetics during pregnancy

There is very limited data in humans regarding AED blood levels and AED clearance during pregnancy. Clearance variability in pregnancy may result in increased risk for seizures that could have detrimental effects on the mother and fetus. Further differences in AED blood levels may affect risk for behavioral deficits since teratogens typically act in a dose-dependent manner. In humans, AED blood levels should be tested for different AEDs and for different polytherapies.

### 4 Genetics

Teratogens act on a susceptible genotype. This may explain much of the individual variability observed after fetal AED exposure (Finnell et al., 1987). An understanding of the underlying genetic risk factors could lead to improved prediction of risks. Further, such knowledge could suggest potential avenues for treatments to prevent or reverse adverse AED effects.

### 5 Preventive or ameliorating therapies

A better understanding of the mechanisms underlying the observed AED effects is needed in order to develop ways to block the effects of AEDs on the immature brain and to treat the child after deficits have already occurred. Some possibilities of future treatments could include vinpocetine that has been shown to improve neuronal plasticity in rats, or estradiol that may ameliorate neurotoxicity of some of the AEDs (see below).

Because the vulnerability of the immature brain to AED effects likely extends beyond birth in humans and since AEDs are used in neonates to treat seizures, studies are needed to examine the risks of neonatal AED exposure. Pharmacological blockade of NMDA or activation of GABA(A) receptors, blockade of voltage-dependent sodium channels, and oxygen-induced widespread apoptotic neurodegeneration during the so-called brain growth-spurt period in rodents. Because such measures are often necessary in pregnant WWE, as well as infants and toddlers, searching for adjunctive neuroprotective strategies is warranted. AEDs have been shown to reduce neurotrophins and signal proteins for cell growth. 17 β-estradiol is a neurotrophin and ameliorates neurotoxicity of drugs that affect the infant rat brain in the fashion noted above. 17 β-estradiol and related compounds may be neuroprotective agents suitable for use in the fetus and critically ill infants and toddlers exposed to AEDs. Its supplementation may lead to improved neurocognitive outcomes in these populations (Asimiadou et al., 2005).

Vinpocetine (ethyl apovincaminate) discovered during the late 1960s has been used in the treatment of central nervous system disorders of cerebrovascular origin. The increase in the regional cerebral blood flow in response to vinpocetine administration is well established. Vinpocetine is also a phosphodiesterase type 1 inhibitor, which has been shown to enhance long-term potentiation and improve memory in animal models (Filgueiras et al., 2010). Vinpocetine also has anticonvulsive properties (Sitges et al., 2011). Could this medication be applied to treat the cognitive deficits seen from fetal alcohol syndrome or AED exposure?

Alcohol exposure in the immature brain leads to a persistent disruption in ocular dominance plasticity in an animal model. Using a combination of electrophysiological and optical imaging techniques, one animal study showed that vinpocetine, restores ocular dominance plasticity in the ferret model of fetal alcohol exposure (Medina et al., 2006). This finding could contribute to a possible treatment of cognitive deficits associated with cognitive disorders such as fetal alcohol syndrome in humans. Another study done in Paul and Medina (2012) using the ocular dominance ferret model showed that the plasticity deficit can be reversed if the previously alcohol-exposed animals are treated with a therapy that has similar effects to the downstream effects of a phosphodiesterase inhibitor like vinpocetine. Vinpocetine’s mechanism of action is thought to act by inhibition of phosphodiesterase type 1 and may increase cAMP and cGMP levels, activating transcription factors such as the cAMP response element binding protein (CREB) and the serum response factor (SRF). SRF is important for many plasticity processes such as long-term potentiation, long-term depression, spine motility, and axonal pathfinding Immature ferrets were exposed to alcohol and later treated during the period of monocular deprivation using a Sindbis viral vector to express a constitutively active form of SRF in astrocytes. Using single-unit recordings and optical imaging of intrinsic signals, the investigators found that overexpression of a constitutively active form of SRF restored ocular dominance plasticity in the alcohol-treated animals. This finding suggests that overexpression of SRF may reduce the deficits in neuronal plasticity seen in models of fetal alcohol syndrome. It therefore follows that a medication like vinpocetine that has downstream upregulation of SRF may have the same effect on neuronal plasticity.

A study in rats supports the ability of vinpocetine to reverse alcohol-induced deficits (Filgueiras et al., 2010). The Morris maze test was used to assess whether vinpocetine restores learning performance in rats exposed to alcohol during the third-trimester equivalent of human gestation. Early alcohol exposure significantly affected the performance to find the hidden platform as the ethanol-exposed rats took significantly longer than the control group. However, when the alcohol-exposed animals were treated with vinpocetine their performance was restored to control levels. The results show evidence for the potential therapeutic use of vinpocetine in fetal alcohol syndrome and suggest that a phosphodiesterase type 1 inhibitor can improve learning and memory deficits in rats exposed early to alcohol (Filgueiras et al., 2010).

## Summary

Animal studies have demonstrated that exposure of the immature brain to AEDs can produce neuronal apoptosis, impair physiology in surviving neurons, and result in reduced cognitive outcomes. However, only a limited number of AEDs have been tested in these animal models. There are no human studies examining the effects of fetal AED exposure on neuronal apoptosis and electrophysiology of the remaining neurons. Human studies have demonstrated impaired cognitive outcomes in children exposed *in utero* to valproate, which occurs in a dose-dependent manner. There is some human data providing evidence for adverse cognitive effects of phenobarbital and for absence of major adverse effects for carbamazepine, lamotrigine, and levetiracetam. Evidence for other AEDs is inconclusive or non-existent in humans.

Even in animals, the full extent of mechanisms are poorly understood. Our knowledge in humans is very inadequate in regards to the reasons for individual variability, the exact developmental window of vulnerability to AEDs after birth, and potential approaches to blocking or mediating adverse effects. Thus, there are many areas that critically require additional research.

## Conflict of Interest Statement

Dr. Gedzelman reports no potential conflicts of interest. Dr. Meador reports receiving research support from the GlaxoSmithKline, EISAI Medical Research, Myriad Pharmaceuticals, Marinus Pharmaceuticals, NeuroPace, Pfizer, SAM Technology, Schwartz Biosciences, and UCB Pharma, the Epilepsy Foundation, and the NIH; received salary support to Emory University from the Epilepsy Consortium for research consultant work related for NeuroPace, Novartis, Upsher-Smith, and Vivus; served as a consultant for Eisai, GlaxoSmithKline, Johnson and Johnson (Ortho Mc Neil), Medtronics Spherics, and UCB Pharma, but the monies went to a charity of the company’s choice; received travel support from Sanofi Aventis; and also serves on the Professional Advisory Board for the Epilepsy Foundation and the editorial boards for Cognitive and Behavioral Neurology, Epilepsy and Behavior, Neurology, and Journal of Clinical Neurophysiology.
